# Trust in Government or in Technology? What Really Drives Internet Voting

**DOI:** 10.1177/10659129251321424

**Published:** 2025-02-18

**Authors:** M. Belén Abdala, Carolina Plescia, Ming M. Boyer, Anna Lia Brunetti

**Affiliations:** 1 27258 University of Vienna, Vienna, Austria; 2 1190 Vrije Universiteit Amsterdam, Amsterdam, Netherlands

**Keywords:** internet voting, turnout, trust, experiment, political behavior, voting modalities

## Abstract

Internet voting is considered a crucial potential technological innovation, and scholars agree that trust plays a key role for its adoption and use by citizens. But which type of trust is essential, trust in government or trust in technology? We leverage on a cross-sectional analysis and a preregistered online experiment in Estonia to test a multidimensional trust framework. Examining the impact of trust dimensions on i-voting likelihood, we unveil a robust correlation between trust in government and in i-voting technology. While both elicit the intention to i-vote, trust in technology emerges as a stronger driver for the decision between online or in-person voting. These findings significantly contribute to comprehending i-voting and offer insights into the practical implementation of technology in democratic processes.

## Introduction

In response to the steady decline in electoral participation (Kostelka and Blais 2021), politicians and political scientists have identified the use of digital voting technologies as potential solutions. In particular, forms of balloting that allow people to vote at a time and place different from Election Day are often expected to foster turnout (Petitpas, Jaquet, and Sciarini 2021). As such, governments across the globe have actively engaged in pilots testing internet voting (i-voting), a voting modality in which individuals cast their votes over the internet (Goodman and Stokes 2020). However, the scope and success of these pilots has varied greatly (Turnbull-Dugarte and Devine 2023). Many of the countries that have experimented with i-voting (e.g., the US, UK, Australia, France, the Netherlands, Finland, Spain, and Greece) discontinued these pilots following varied outcomes in terms of voter turnout, alongside other concerns related to system security and trust (Goodman and Stokes 2020; Lust 2018).

In places where i-voting continues to be practiced (e.g., in Estonia and Switzerland), research indicates that trust is the strongest predictor for citizens’ decision to i-vote (Trechsel and Vassil 2011). However, it remains an open question which type of trust is decisive for i-voting—trust in government or trust in technology. In some studies, trust in i-voting technology (Nemeslaki, Aranyossy, and Sasvári 2016) or in the internet (Powell et al., 2012), are key in explaining the intention to vote online. In other works, trust in government is what matters (Carter and Bélanger 2005), while in certain studies, trust in the government is deemed unimportant (Powell et al., 2012). These effects are difficult to disentangle given that trust in government and trust in technology can be interrelated (Germann and Serdült 2017; Goodman and Stokes 2020). So, which type of trust is essential for i-voting?

To disentangle trust in government and trust in i-voting technology and evaluate their effect on i-voting, we propose and test a framework in which trust is a multidimensional construct. In such a framework, public confidence in i-voting involves a trustor that is willing to give their trust (i.e., citizens), and two elements to which this trust is addressed: a trustee that provides the service and generates this trust (i.e., the government, state, or institutions organizing the elections) and an intermediary that is used to contribute to a certain outcome (i.e., the i-voting technology itself) (Lippert and Davis 2006). As such, the different dimensions of trust are related but can have separate effects on the likelihood to i-vote. We test this framework: we run a survey collecting original cross-sectional data and a preregistered, novel online experiment aimed at disentangling the effect of these two dimensions of trust (*N* = 1,492). The testing case is Estonia, the only country currently using i-voting for all national-level elections. This feature enables us to assess the influence of each trust component on the likelihood of i-voting when individuals have the option to choose between online and in-person voting.

We find that trust in i-voting technology and trust in government are strongly related and both impact the likelihood to i-vote. However, the levels of trust in i-voting technology have a larger influence on i-voting. As a consequence, excluding citizen trust in i-voting technology from empirical analyses inflates estimations regarding the impact of trust in government on i-voting. Additionally, their effects on in-person voting differ significantly. Trust in i-voting technology reduces in-person voting, forming a displacement effect. In contrast, trust in government has no discernible effect on in-person voting, such that the increase in i-voting consists of additional voters.

Our contribution is three-fold. First, to address the mixed findings on the link between trust and i-voting (Lippert and Ojumu 2008; Lust 2018), we examine the various conceptualizations of trust used in previous work and theoretically derive a single conceptualization of trust specifically geared towards i-voting—consisting of trust in the government and trust in the i-voting technology. Second, we empirically test the impact of these two elements of trust on the decision to i-vote in an upcoming election, dialoguing with previous works that examine the role of internet voting in turnout (Goodman and Stokes 2020). Third, we contribute to the scarce set of experimental evidence on the link between trust and i-voting by testing our hypotheses in both a cross-sectional and an experimental manner (Turnbull-Dugarte and Devine 2023). As a result, this paper contributes to long-standing discussions about the consequences of voting modalities as well as with broader debates about the role of technology in democracies, and its link to both trust and electoral participation.

## Internet Voting

Voting can be costly. One has to spend time, effort and sometimes money to travel to a polling station and cast a ballot. I-voting, a voting modality in which citizens can cast ballots remotely through an internet connection, is one of the most discussed possibilities to reduce such direct *costs* of voting (Goodman and Stokes 2020; Santana and Aguilar 2021).

As a result, by the early 2000s i-voting was considered a promising solution to low turnout and governments around the world decided to test the use of internet ballots both at the national and local level (Lust 2015, 2018). However, more than 20 years after the initial experimentation, the effects of i-voting on voter turnout remain unclear. Some studies find large positive effects among disengaged citizens (Vassil et al., 2016), as well as younger and more educated voters (Solop 2001). For example, Goodman and Stokes’ (2020) research on the local elections in Ontario showed that i-voting increased turnout, especially when voting by mail was not available and registration was not required. But other studies find null or even negative effects of i-voting on turnout (Bochsler 2011). An example of this is the work of Germann and Serdült (2017) on Switzerland, which indicates that the introduction of i-voting did not raise voter turnout in federal referendums in Geneva and Zurich where this modality was available alongside postal and in-person voting. This evidence is also in line with various governments’ analyses in which the absence of a sharp increase in turnout was cited as a major factor in pilot program cancellations in the UK, Norway and Austria, for example, (Germann and Serdült 2017; Goodman, Pammett, and DeBardeleben 2010). In addition to the unclear impact on turnout, some reports also consider security concerns and shrinking electoral budgets as key factors for the reduced adoption and use of i-voting (Alvarez and Hall 2004). Nevertheless, even if internet voting is still rarely offered to resident voters, it is more common when it comes to expatriate voters (e.g., France, some states in U.S. and Switzerland) (Germann and Serdült 2014) and continues to be practiced in several parts of the world (Turnbull-Dugarte and Devine 2023).

Considering the ongoing advances in digital technologies and its impact on liberal democracies in terms of political communication, political participation, and policymaking (Gilardi 2022), it seems likely that elections will undergo some degree of digitization. Hence, exploring the factors that shape the adoption and utilization of digital technologies in elections is of growing importance. Indeed, understanding user acceptance, adoption, and actual usage of modern technology has become a rich stream of research in itself (see Granić (2024) for a full discussion). The most widely used model, the Technology Acceptance Model (TAM), posits that user behavior towards technology is influenced by perceptions of usefulness and ease of use (Nemeslaki, Aranyossy, and Sasvári 2016). Although ease of use is crucial for internet applications, it is a necessary but insufficient condition (King and He 2006).

Previous works on i-voting specifically have studied the adoption of technology (and its evolution across time) by looking at who uses this type of alternative voting modality compared to traditional in-person forms of balloting (Bowler and Donovan 2018; Vinkel and Krimmer 2017). Various sociodemographic variables, such as age or digital literacy, and different attitudinal factors, such as political interest, institutional trust, or the more general propensity to trust, have been found to predict support for the introduction of technologies across the electoral process and for remote voting specifically, albeit with mixed results (Lust 2018). For example, while younger voters are generally expected to be more receptive to new opportunities offered by the internet since these have been part of their political socialization, Plescia, Sevi, and Blais’ (2021) study on voting modalities in the U.S. finds that young respondents have an equally high probability of choosing to vote at the polling station or via the internet. As such, not everyone is prone to adopt i-voting and trust appears to be one of the most frequently cited determinants of internet voting. Yet, surprisingly, it still lacks systematic empirical research.

### The Role of Trust in Internet Voting

Our theoretical approach focuses on the role of trust in explaining online electoral participation. Previous work has suggested that low levels of trust lead to less internet voting: citizens have less incentive to i-vote when, for example, they do not trust that the votes will be recorded and counted fairly (Vassil et al., 2016). However, trust has been conceptualized differently across the field (Belanche et al., 2014). While some studies concentrate on the extent to which individuals think that the voting technology is predictable, reliable, and useful (Lippert and Davis 2006), others look into people’s general trust in the internet (Carter and Campbell 2011), their trust in the government (Vassil and Weber 2011), and in the government’s provision of electronic services (Belanche et al., 2014), or the role of culture and citizens’ individual disposition to trust (Warkentin et al., 2018) as key predictors of the use of internet voting. Perhaps for this reason, the investigation into the relation between trust and internet voting has led to mixed findings. Some studies find that trust in government is a strong and significant predictor for i-voting (Carter and Bélanger 2005); others find null results for the effect of trust in government on the intention to i-vote (Powell et al., 2012). Similar patterns have been described for trust in technology: whereas trust in elements related to the implementation of i-voting (e.g., trust in the i-voting technology or related digital technologies more broadly) appear to exhibit a positive correlation with its use (Nemeslaki, Aranyossy, and Sasvári 2016; Warkentin et al., 2018), results vary for studies focusing on more specific items such as trust in the accuracy, security, usability and validity of i-voting technology (see Zhu, Azizah, and Hsiao (2021) for an overview of these studies).

Although trust is conceptualized differently across the field (Belanche et al., 2014), there is consensus on its meaning: trust is relational and involves individuals making themselves vulnerable to other individuals, groups, or institutions (Levi and Stoker 2000). In an effort to systematically define the concept of trust in relation to i-voting, we take as a starting point Rotter’s (1971) definition which understands trust as the belief in the reliability of promises made by others including trust in institutions and technology. This conceptualization is grounded in social learning theory, which proposes that trust is a learned concept influenced by the experiences individuals have with promises. Individuals are argued to form their beliefs about trust by observing whether promises are either fulfilled (positive reinforcement) or broken (negative reinforcement) during social interactions. In turn, this can influence their own attitudes and behaviors in relation to promises made to them. As a result, social learning theory also explains how people might have varying levels of trust in different people, groups, or institutions, when their (perceived) experiences with promises kept or broken differ between them.

This conceptualization of trust requires the presence of certain elements, related both to the individual(s) that are willing (or not) to put their trust in others and to the targets of this trust (Belanche et al., 2014; Shapiro 1987). The first element therefore is the trustor, that is, the individual that gives (or not) their confidence to others (Zhu, Azizah, and Hsiao 2021). In the case of internet voting, the trustors are citizens or potential voters, the individuals that have to make the decision on whether they want to i-vote or not. The second element is the trustee, that is, the entity or organization providing the service. In this paper, we look at the government as key trustee (Belanche et al., 2014). Therefore, our focus is on a form of political trust, where individuals trust the ability and willingness of a core political institution (i.e., the national government) to ensure the organization and administration of fair elections (Levi and Stoker 2000).

**Figure 1.**
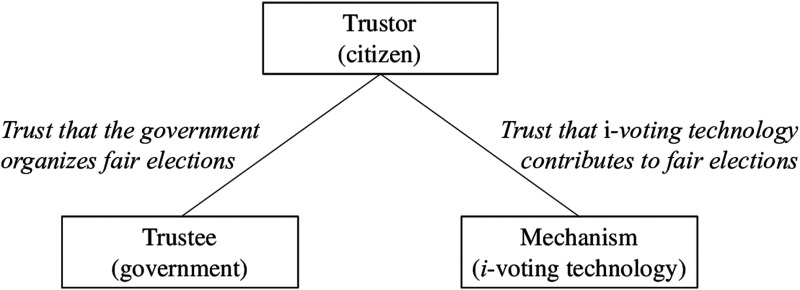
Elements of trust.

Besides these two key elements, Rotter’s (1971) framework allows for a third factor which is sometimes necessary to establish trust, linking the trustor and the trustee by enabling the trustee to fulfill their promise. In the case of internet voting, the third element is the technology that is used to provide the promised service. Technological trust has been defined as an individual’s willingness to be vulnerable to a technology, a willingness that is based on their belief that technology is predictable, reliable, and useful (Lippert and Davis 2006). In other words, it refers to individuals’ trust in the security measures, safety nets and performance structures of this technology (Ryan et al., 2009). This element is distinct from the trustee. The institution responsible for an election can use the best technology available to them and individuals can indeed trust the institution is taking every measure to conform to fair electoral standards. Conversely, citizens can trust technology but simultaneously distrust the intentions and/or capacity of the government responsible for its implementation. Or they can overall trust the government’s intentions but distrust the technology because they perceive it as unreliable and fraudulent, since it assumingly does not guarantee a fair vote count or is susceptible to the influence of external actors. We thus argue that the elements that compose trust are theoretically distinct: the crux of our argument is that to understand the role of trust on i-voting one must simultaneously consider the trustor’s views (citizens’ perceptions) about the trustee (government) and the mechanism (the i-voting technology) that will both influence how likely they are to cast a vote online. Our underlying argument is illustrated in Figure 1.

#### Hypotheses

We have two pre-registered hypotheses.^1^ Our first hypothesis is about the link between the trustee (government) and the mechanism (the voting technology). Previous studies have shown that trust in the government has direct political consequences (Chanley, Rudolph, and Rahn 2000). Distrustful citizens are less likely to vote, but when they do turn out, they are more likely to support the opposition and third-party candidates, as they tend to evaluate more severely the performance of political actors and institutions, such as the President and Congress in the U.S. (Hetherington and College 1998). Recent studies on the response to COVID-19 show that trust in government matters even more than trust in health authorities for citizens’ compliance with safety measures and restrictions (Kritzinger et al., 2021; Nielsen and Lindvall 2021). Hence, we argue that higher trust in the government leads to a higher chance of i-voting. Trusting the government means trusting its ability and willingness to provide services to its citizens, including the capacity to organize elections and to guarantee the elections are conducted safely, equitably, and fairly. Those who trust the government are therefore more likely to i-vote if the possibility to i-vote is available. The first hypothesis states:

H1 Higher levels of trust in government lead to more i-voting.

Our second hypothesis focuses on the mechanism through which the i-voting service is provided. Citizens’ beliefs in how well and how accurately technology functions has been validated as important in users’ adoption of new technologies and citizens’ intentions to use internet voting (Zhu, Azizah, and Hsiao 2021). We follow this literature and argue that i-voting is less attractive when individuals think that the i-voting technology is unpredictable or unreliable. On the contrary, if voters trust that their votes will be accurately recorded and protected from tampering (e.g., designed with transparent processes, robust auditing mechanisms, and clear verification procedures), this can provide reassurance that all votes will be counted fairly and that any irregularities can be identified and addressed. In this case, we expect that voters are more comfortable using internet voting methods. The second hypothesis states:

H2 Higher levels of trust in the i-voting technology lead to more i-voting.

However, it is important to mention that the evidence on the effects of each dimension of trust is so far inconclusive, as trust has not been measured consistently as a multidimensional construct in studying its effect on i-voting (Lippert and Davis 2006). Therefore, it is not possible to pose a formal hypothesis comparing the effects of each dimension of trust on the inclination to i-vote. Hence, we preregistered a research question asking which of the two elements of trust in i-voting drives its use more strongly:

RQ1 To what extent does trust in the government, or trust in the i-voting technology increase the probability to i-vote more strongly?

## Data, Methods, and Case Selection

We test our theoretical proposition in the context of the election for the Estonian Parliament in March 2023. Estonia is a fitting case for our purpose because of the country’s long-lasting experience with i-voting. The country has featured a remote i-voting method since 2005 and is one of the only countries worldwide offering internet voting to the entire electorate for all nationwide, binding elections (Serdült et al. 2015). Unlike ongoing pilot projects in other countries, Estonia’s internet voting technology, along with its rules and procedures, has remained relatively stable since then and has generally received support from the country’s major political parties (Alvarez, Hall, and Trechsel 2009). Although partisan conflict over internet voting has emerged at times, opposition has diminished as the practice has become more widespread and as different parties have alternated in governing roles (Ehin et al. 2022). In Estonia, this voting modality allows voters to cast their ballots from any computer with internet access, and is available before Election Day, during a designated pre-voting period, in which voters can log into the system using their electronic IDs and cast a ballot. Voters may cast their internet ballots multiple times, and only the last one is considered as valid for the official tally. Various paper ballot options are also available before and during Election Day. Any paper ballot cast in the voting period will be counted, canceling any internet ballot cast by the voter. The number of internet voters in Estonia has been rising steadily since its early implementations (Vassil et al. 2016), from less than 6 percent of eligible voters in the 2005 national elections to 50 percent of votes in 2022.

Focusing on Estonia allows us to study the role of trust in a country where people have already been confronted with i-voting, ensuring that the novelty of the voting modality does not affect the results. This is crucial because new voting modalities can trigger various reactions, including skepticism, curiosity, or even resistance, which make it difficult to manipulate “trust” and obscure its effects over the decision to vote or not, and how to do so. By selecting Estonia, we can isolate and analyze the impact of trust more accurately, free from the potential confounding effects of novelty. Estonia has a relatively large proportion of the population that trusts the government (40.33 percent). However, while this level of trust is higher than in the U.S., it remains below the average for European countries.^2^ The data collected by the Eurobarometer in 2022 shows that Estonians tend to trust the internet more than the EU-27 average albeit differences are relatively modest.^3^

As described below, we run two separate studies: one cross-sectional analysis of Estonian citizens investigating their real-world attitudes and one experiment in which they evaluate a fictional country to further a causal argument. Data for both studies were collected in the same online survey by the survey company Norstat 2 months before the election (between 3 and 17 January 2023); we chose this timeframe for fieldwork to reduce the effects of campaign specifics on trust or voting intentions. The sample of respondents is nationally representative on age, gender, education, and region—and large enough to detect small effects (see also below). Appendix A provides details on the ethical aspects of the study, while Appendix B show the data quality and how well the sample matches the population of Estonia.^4^

## Study 1: Cross Sectional Study

### Research Design

Study 1 uses cross-sectional data, with a sample of *N* = 1,492 respondents.^5^ To capture respondents’ intended voting modality in the upcoming general election, we asked participants “*How would you prefer to cast your ballot in the upcoming Riigikogu Elections?*” The answer options cover all possible voting modalities in Estonian general elections namely: (1) pen-and paper *voting* at a *polling* place on election day, (2) pen-and paper *voting* at a *polling* place before election day, (3) online via internet voting and (4) postal voting. The dependent variable in our study is a dummy taking a value of 1 for respondents choosing option 3 and 0 for all other voting alternatives.^6^

We have two main independent variables. First, trust in government is measured using the following question: “*Please rate how much trust you have in [the government] on a scale from 0 to 10, where 0 means ‘no trust at all’ and 10 means ‘complete trust’*.” Second, trust in the voting technology measured by asking “*How high from 0 to 10 is the risk of fraud in internet voting?”, being 0 “low risk of fraud in internet voting” and 10 “high risk of fraud in internet voting.”* The measurement was later recoded so that higher numbers represent higher levels of trust (i.e., less perceptions of risks of fraud). This approach to measure trust in the voting technology was based on previous works that consider perceptions of the risk of fraud as a major component of this type of trust, which is different from other elements that could explain individual preferences for i-voting, such as, its usability (Alvarez et al., 2021). Trust in government and trust in i-voting correlate quite strongly (*r* = .62).

We control for variables that the literature on turnout and voting behavior has shown to affect the decision to vote and that could act as contending mechanisms. These are: age, gender, education, and political interest. Age is measured in years. Gender is recoded as a dichotomous variable that takes value 1 for women and 0 for men.^7^ Education is captured as the level of achieved education, where higher values represent higher levels of education. Political interest is a categorical variable from not at all interested (1) to very interested (4). We also control for propensity to trust, trust in political parties, and the time spent online as a proxy for digital literacy. Propensity to trust is captured by asking people *“Generally speaking, would you say that most people can be trusted, or that you can’t be too careful in dealing with people? Please answer on a 0 to 10 scale, where 0 means you can’t be too careful and 10 means that most people can be trusted.”* Trust in political parties is measured on a 0 (no trust at all) to 10 (complete trust) scale. The inclusion of both indicators for general trust and trust in parties allows us to control for other sources of trust that are external to the voting process itself and could serve as confounders, as some people could generally be more trustworthy than others. Finally, time spent online is measured as a continuous variable stating the number of hours a person spends online on a typical day. We include an additional model with a subjective measure of income, assessed on a 4-point scale from low (finding it very difficult to manage on current income) to high (living comfortably on current income). As education and income are often correlated, the results for models incorporating this variable, which align with all other models, are provided in Table C2, Appendix C.

Given the dichotomous operationalization of the outcome variable, we use logistic regression models and report results as predicted probabilities.

### Results

Table 1 presents the logistic regression results for our dependent variable, “preference for i-vote compared to in-person voting,” that takes value 1 for internet voting and 0 for any form of in-person voting. Model 1 examines trust in government as the main independent variable, Model 2 focuses on trust in i-voting, and Model 3 includes both variables.^8^

**Table 1.** Predicting I-Voting With Trust in Government and Trust in I-Voting.

	Model 1	Model 2	Model 3
Trust government	0.31***		0.06
	(0.03)		(0.04)
Trust i-voting		0.36***	0.34***
		(0.02)	(0.03)
Female	−0.19	−0.22	−0.24
	(0.13)	(0.15)	(0.15)
Age	−0.02***	−0.02**	−0.01**
	(0.00)	(0.00)	(0.00)
Education	0.18***	0.18***	0.17***
	(0.04)	(0.04)	(0.04)
Political interest	−0.37***	−0.40***	−0.39***
	(0.08)	(0.09)	(0.10)
Trust in political parties	−0.11**	−0.01	−0.05
	(0.04)	(0.04)	(0.04)
General propensity to trust	−0.05	−0.05	−0.05
	(0.03)	(0.03)	(0.03)
Time spent online (daily)	0.07**	0.06*	0.06*
	(0.02)	(0.02)	(0.02)
Constant	0.77*	−0.27	−0.30
	(0.33)	(0.38)	(0.38)
Observations	1385	1268	1263
LR chi2 (9)	281.76	475.54	478.14
Pseudo R2	0.1577	0.2918	0.2944

*Note*. Standard errors in parentheses: *p < 0.05; **p < 0.01; ***p < 0.001.

**Figure 2.**
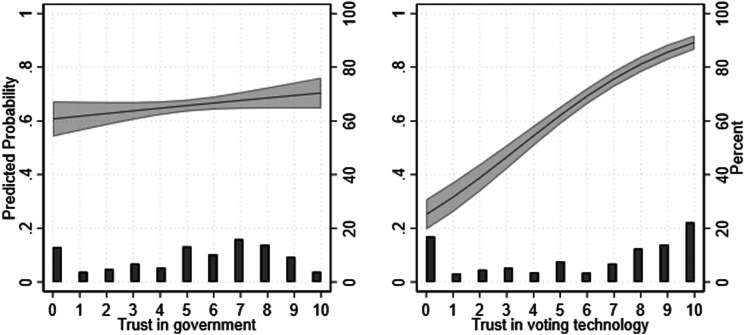
Predicted probabilities and 95 percent CI of *i*-voting compared to in-person voting, over trust in the government and trust in internet voting technology, controlling for each other (Model 3). *Note*: The right axis represents the histogram of the levels of trust as percentages.

These results support our hypotheses, as they indicate that both types of trust are positively linked with i-voting. Figure 2 additionally shows the predicted probabilities of an increase in government trust and in online voting, respectively (Model 3), as well as a histogram of the levels of trust as percentages, which are in line with the previously discussed averages for Estonia.

The mean predicted probability of preferring internet to in-person forms of voting is of 0.61 if one does not trust the government at all (trust in government = 0) and increases to 0.70 when government trust is high (trust in government = 10), averaging across the sample values of all the other control variables. While the effects of both types of trust are related to (some) increase in i-voting, the increase for trust in i-voting is stronger: the mean predicted probability of i-voting is 0.25 when the level of trust in i-voting is at its lowest (0) and increases to 0.89 when it is at its highest possible score (10). This is a much steeper increase compared to trust in government, as visualized in Figure 2.

When it comes to our research question, the results show that trust in i-voting has a stronger effect on i-voting than trust in government. What is more, including only trust in government in an analysis predicting i-voting (as we did in Model 1) can produce misleading findings, as the effect of trust in government is much stronger (and significant) without the inclusion of trust in i-voting. In other words, a lack of trust in government only seems to lead to less i-voting insofar as it is related to a lack of trust in the i-voting technology, while a lack of trust in i-voting technology is related to decreased i-voting regardless of trust in government. This is confirmed by a Wald test, performed to compare the coefficients for trust in the government and in the internet voting technology. Results indicates a highly significant difference between the two (χ^2^ = 19.39, *p* < 0.001), indicating that the relative impact of trust in technology on i-vote is significantly larger than that of trust in government.

Overall, these results provide evidence that both types of trust are interrelated, but it is trust in the internet voting technology that has a greater and independent impact on i-voting. While trust in government is related to i-voting as well, its impact is largely explained by trust in the i-voting technology. However, we cannot exclude the possibility that the correlations between i-voting and either type of trust are confounded by a third variable. We address these issues in an experiment to further investigate the role of multidimensionality of trust in explaining i-voting.

## Study 2: Experimental Study

### Research Design

The goal of our experiment is to test (1) whether an increase (decrease) in trust in government and in i-voting technology causes an increase (decrease) in i-voting intention; and (2) which aspect matters the most, trust in government or in the i-voting technology. To analyze the impact of trust in i-voting on electoral participation at the individual level, we conducted a 2 (trust/distrust) × 2 (government/voting technology) + control experimental design.

The experiment again has a sample size of *N* = 1,492 respondents.^9^ Of this experimental sample, roughly 300 participants were randomly assigned to each group. Overall, we observe no large significant imbalances between groups, suggesting that the random assignment led to an even spread of respondent characteristics across the conditions (see Appendix B for more information about the sample distribution).

#### Treatment Conditions: (Dis)trust Voting Technology and (Dis)trust Government

The treatment was created using a vignette that included specific cues about trust in the government or in the i-voting technology. In the experiment, we exposed participants to one of five descriptions of a fictional country called Mancosia. In the control condition, participants read only a short paragraph that describes the size, location, and implementation of electronic government procedures of the country. In the other four experimental conditions, participants additionally read a description of either the government or the internet voting technology of Mancosia. In the (dis)trust government conditions, the country has a stable (unstable), democratic (oligarchic) government, with regular (seldom) referenda and a good (bad) reputation with the public who are satisfied (dissatisfied) with and trust (distrust) the government. The goal is to underline how the government works in relation to the political system, the elites and the country’s citizens, as well as how it is perceived by citizens. In the dis(trust) voting technology conditions, voting legislation is quite (not) transparent, and public authorities are very (not) service oriented. There is a large (small) budget for information technology that has positive (negative) repercussions on trust in e-government systems. The goal is to describe how the voting technology works, in relation to its regulation, goals and implementation, as well as how it is perceived by citizens.

By manipulating both dimensions of trust separately we can disentangle which dimension of (dis)trust (if any) is guiding the effect. The hypothetical set up with a fictional country is crucial since it allows us to manipulate and measure the attitudes of our respondents towards the government and i-voting technology regardless of their pre-existing views. After the experiment, participants underwent a thorough debriefing. Appendix D includes full information on the preregistration plan for the experiment, the wording of the stimuli and questions, as well as an overview of the distribution of sociodemographic and political variables in each group.

To determine whether the manipulation worked, after exposure to the treatment we asked respondents to imagine being citizens of Mancosia and answer how much they would trust their government and the online services it provides, on a 0 (no trust at all) to 10 (complete trust) scale. We performed ANOVAs and post-hoc tests (pairwise comparisons) to estimate the effects of a change in the level of trust in the government or in online services, comparing each treatment group to the control group. Results indicate that the manipulations worked as planned. Regarding the (dis)trust voting technology condition, there is a positive and statistically significant difference in trust between the group who was exposed to the trust voting technology condition and the control group (ΔM = 0.83, t = 3.41, *p* = 0.001), as well as a negative and significant difference between the distrust voting technology condition and the control group (ΔM = −3.65, t = −15.32, *p* < 0.001). The same is true for the contrast between the trust (ΔM = 1.48, t = 6.22, *p* < 0.001) and distrust (ΔM = −3.69, t = −15.13, *p* < 0.001) government conditions, compared to the control group. It is important to note that there were spillover effects of one dimension of trust to the other. The trust government conditions are also associated with increases in trust for i-voting and vice versa. This is in line with the cross-sectional analysis of Study 1, indicating that both types of trust are related. See Appendix E for the full results.

#### Dependent Variable: Likelihood to (i-)vote

The main outcome variables of our study are the likelihood to vote online or in-person, on a 0 (very unlikely) to 10 (very likely) scale. To measure this, after exposure to the stimulus material, we showed respondents the following statement: “*Imagine again that you are a citizen of Mancosia and that the country is holding elections in the upcoming weeks. Besides voting in person at a polling station, Mancosia allows online voting via the internet*”. We then asked them to state, separately, how likely they would be to vote online via the internet or in person at a polling station. The order of these questions about the voting modalities was randomized.

Since the dependent variables (the likelihood to vote in person or via the internet) are continuous and the main independent variable refers to the treatment exposure, we use ANOVA models to test the hypotheses. Accordingly, we carry out contrasts to compare each experimental condition to the control group as well as contrasts between the 4 experimental conditions: trust i-voting; distrust i-voting; trust government; distrust government. Mean scores on the dependent variables in the control condition are used as comparison.

### Results

**Figure 3.**
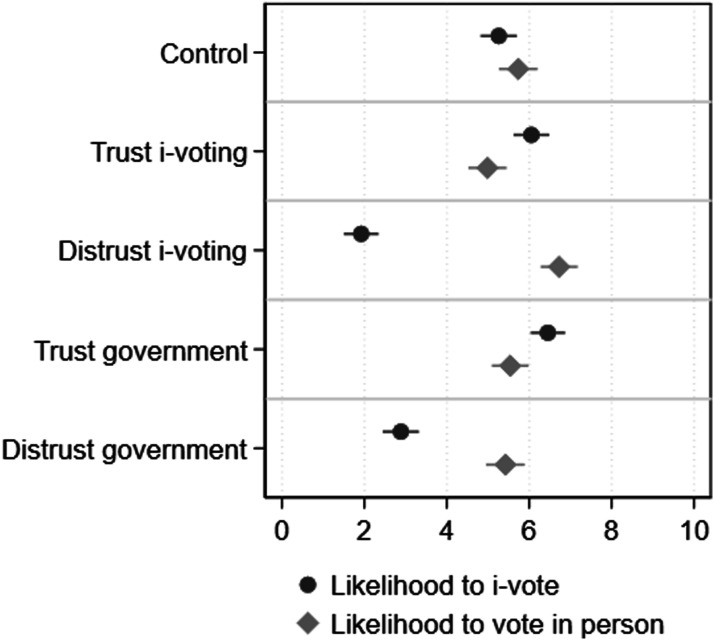
Estimated marginal means and 95 percent confidence intervals of i-voting and in-person voting per condition. *Note*: Effects calculated as pairwise comparisons of means with equal variances, using Tukey’s adjustment.

Figure 3 presents the estimated marginal means of internet and in-person voting (measured using the 0–10 continuous variable) for people exposed to each experimental condition and the control group. The full results can be found in Appendix F. The results show a statistically significant difference between groups in terms of both i-voting (*F* (4,1299) = 86.12, *p* < 0.001) and in-person voting (*F* (4,1299) = 7.80, *p* < 0.001). The control group is used as a baseline in the contrasts below. In line with our expectation and the cross-sectional analysis, the models indicate that the likelihood to vote online is reduced for respondents in the distrust conditions of both the voting technology and the government, compared to those in the control group. The probability to i-vote decreases by 3.34 points for respondents in the distrust voting technology condition, and by 2.37 points for those in the distrust government condition, compared to the control group. The results are statistically significant at the 0.001 level. Alternatively, being exposed to positive views and messages about either i-voting technology or the government increases the likelihood of i-voting, albeit with a smaller magnitude, as exposure to the trust voting technology condition is linked to an increase of 0.79 point (t = 2.54 and *p* = 0.08), and to the trust government condition to an increase of 1.19 points (t = 3.41 and *p* = 0.001) in the probability of i-voting. Overall, the strongest effects are observed in the distrust treatments. This may be because Estonians, having repeatedly been exposed to positive information about and experiences with i-voting, might tend to assume a trustworthy government and technology unless informed otherwise. As a result, they are more sensitive to negative information, which contrasts with their default expectations. This interpretation aligns with the observation that baseline trust in both government and technology is generally high in Estonia.

Turning to the likelihood of in-person voting as the outcome variable, our findings suggest that voting technology distrust is associated with more in-person voting (ΔM = 0.99, t = 3.00, *p* < 0.001), while trust in the voting technology subsequently reduced the likelihood to vote in person (ΔM = −0.75, t = −2.23, *p* = 0.17). Interestingly, the differences between the government distrust and the control condition (ΔM = −0.31, t = −0.92, *p* = 0.89), as well as the distance between the government trust and control condition are not statistically significant (ΔM = −0.19, t = −0.55, *p* = 0.98). It appears that trust or distrust in voting technology and in the body managing the election process are crucial factors influencing the choice to i-vote. However, the level of voting technology (dis)trust itself predominantly determines whether individuals opt for in-person voting or contemplate switching to an alternative voting method. Notably, government trust doesn’t impact the decision to vote in person.

These results therefore reveal an interesting puzzle. Greater trust in voting technology encourages i-voting, while distrust in technology pushes voters towards in-person voting. However, the pattern is different for trust in government: higher trust in government boosts i-voting but has no effect on in-person voting. Essentially, the results are more ambiguous with respect to the relative impact of the two types of trust. Both trust in voting technology and trust in government influence voting methods. Conversely, while distrust in the voting technology boosts in-person voting, government distrust negatively affects i-voting without a compensatory rise in in-person voting, potentially reducing overall turnout. In summary, the experiment highlights that both types of trust are interrelated and strongly correlated. While trust in government is important for overall turnout, the result that trust in the voting technology matters most for i-voting is confirmed.

**Figure 4.**
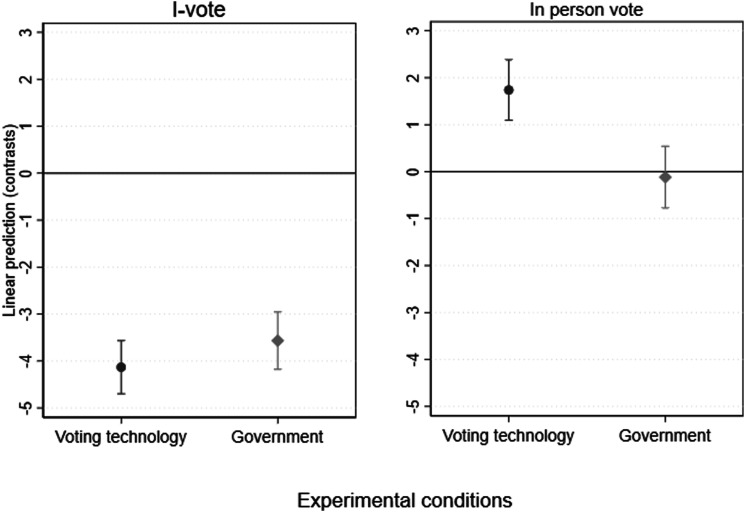
Estimated effects of being exposed to each “distrust” condition compared to the “trust” condition on the likelihood of internet and in-person voting. *Note*: The outcome variables are the likelihood of i-voting and in-person voting (measured on a 0–10 scale where 0 means very unlikely and 10 very likely). The figure shows the effects calculated as contrasts between the distrust and trust manipulations for the i-voting experimental group and the government experimental groups, respectively. Lines indicate 95 percent confidence intervals. Estimates are obtained as margins, pw-comparisons from the ANOVA models presented in Appendix F.

When comparing the high trust condition to the low trust condition for both outcome variables, the results are even clearer. Figure 4 shows the effects calculated as contrasts between the distrust and trust manipulations for the voting technology experimental groups and the government experimental groups, respectively. Results indicate that people exposed to the voting technology distrust condition are −4.13 points less likely to i-vote than those in the respective trust condition (SD = 0.31, *p* < 0.001); while respondents exposed to the government distrust condition are 3.57 points less likely to i-vote than those in the government trust condition (SD = 0.31, *p* < 0.001). If we look instead at the likelihood of voting in person, our findings suggest that respondents who face an increase in their voting technology distrust are 1.74 points more likely to vote in person compared to people who experience more trust (SD = 0.33; *p* < 0.001). Those in the government distrust condition are 0.12 points less likely to vote in person than those exposed to government trust manipulation, although the results are not statistically significant (SD = 0.33; *p* = 0.997).

To test the different effects of trust in the i-voting technology and trust in government on the inclination to i-vote or vote in person, we calculate the interaction effects between the level of trust (low vs high) and the type of trust (i-voting technology vs. government) on each dependent variable. The control condition is excluded from this analysis (see Appendix F for more information). This analysis shows only a direct effect of the level of trust on i-voting, F (1, 1047) = 330.04, *p* < 0.001, but no interaction effect between level and type of trust, F (1, 1047) = 1.78, *p* = 0.182. In contrast to the cross-sectional analysis, this indicates that both types of trust similarly affect the inclination to i-vote. However, the direction of the difference between the conditions is in line with the previous analyses, where trust in i-voting technology has a stronger effect on i-voting than trust in government, and this effect is significant for one-tailed analysis. In contrast, there is a significant interaction effect between the level and type of trust on in-person voting, F (1, 1047) = 15.71, *p* < 0.001, showing that the level of trust in the i-voting technology and the level of trust in the government have different effects on the inclination to vote in-person. While distrust in the i-voting technology increases in-person voting, distrust in the government has no effect on in-person voting.

Lastly, we conducted an additional preregistered analysis, using two additional outcome variables to estimate the level of acceptance of i-voting. These variables measure the level of agreement on a 0–10 scale (strongly disagree–strongly agree) with the extent to which i-voting allows for efficiency and government accountability.^10^ The analysis was conducted using one-way ANOVAS and contrasts comparing each treatment condition to the control group. The results are in line with our hypotheses and preceding findings (see Appendix G). While participants in both high-trust conditions show higher agreement with both statements than participants in the control condition, the results are not significant. Participants in the distrust conditions, on the other hand, show lower and statistically significant levels of agreement with the statements on perceptions of efficiency and accountability of i-voting than those in the control condition. This shows that, in addition to the inclination to i-vote, both types of trust can affect citizens’ views about i-voting.

## Conclusions

Our aim in this paper was to examine the role of trust in the decision to i-vote. In particular, the objective was to disentangle and causally estimate the extent to which a change in the levels of trust in i-voting technology and trust in the government influence online and in-person turnout. Beyond confirming trust as a pivotal factor in understanding i-voting (Belanche et al., 2014; Petitpas, Jaquet, and Sciarini 2021; Warkentin et al., 2018), we make several crucial contributions to long-standing discussions about the effects of convenience voting modalities, trust, and technological developments on political participation.

Our study advances existing models by employing a multidimensional model of trust (Levi and Stoker 2000; Rotter 1971), discerning between trust in the government and trust in i-voting technology. Previous works have indeed highlighted the relevance of trust in explaining the decision to i-vote but have disagreed on which type of trust—trust in government or technology—matters most (Trechsel and Vassil 2011). In contrast, we aimed to disentangle the role of trust in government and trust in i-voting technology, by proposing a framework based on a trustor that is willing to give their trust (i.e., citizens), a trustee that provides the service and generates trust (i.e., the government) and an intermediary that contributed to the provision of the service (i.e., the i-voting technology) (Lippert and Davis 2006).

Testing this model in Estonia has led to three important insights. First, we discovered a strong relationship between the two types of trust, evident in the high correlation observed in our cross-sectional analysis and the spill-over effects seen in the manipulations on both types of trust. The strong correlation between these two variables is understandable, given that a government’s evaluation hinges in part on its proficiency in implementing an electoral system, while an assessment of an i-voting technology depends on the institution responsible for its implementation. Therefore, our findings suggest that attempts to improve one type of trust without improving the other may be futile in an effort to increase i-voting among citizens. This means that, even in countries where citizens have relatively high trust in the government to organize elections, it is not only important to invest in safe and reliable i-voting technology—but also to build trust in these systems among the public. Without such technological trust, the use of i-voting will be severely limited. As this has direct consequences for democracies and how we participate in politics, future work might focus on creating and testing interventions to increase trust in i-voting technology.

Second, our findings reveal distinctive effects between the two dimensions of trust. While distrust is the strongest predictor for citizens’ decision to i-vote, a result that is in line with existing research, the effects of distrust of i-voting technology and distrust of government differ significantly. Trust in i-voting technology has a greater impact on i-voting, surpassing the role of trust in government. In contrast, trust in i-voting technology reduces in-person voting, whereas trust in government seems to have no discernible effect on in-person voting. In other words, there is a replacement effect in which at least some of the positive changes in i-voting are mirrored in a negative change in in-person voting. Trust in government, however, causes an increase in i-voting without a decrease in in-person voting. These results underscore that trust in i-voting technology might be the most important factor influencing citizens’ tendencies to i-vote. On the one hand, while giving people the option to vote online may make voting easier for certain subgroups of the population, the role of convenience voting modalities will be moderate if citizens do not have enough trust in the voting technology—even if trust in government is high. On the other hand, it seems that advancing citizens’ trust in government has a stronger potential for an increase in electoral participation overall than citizens’ trust in the i-voting technology.

Third, the implementation of a multidimensional model of trust revealed that trust in government may only affect i-voting insofar as it is correlated with trust in i-voting technologies. This correlation may be caused by several perceptions. For example, one may believe that a government is illegitimate because it is elected through i-voting or one can believe that the i-voting technology is not trustworthy because it is implemented by a corrupt government. However, trust in government consists of more than the government’s ability to conduct a digital election—and these other parts of trust in government do not seem to be related to i-voting intention. As such, there is a substantial chance that previous work that did not consider citizens’ trust in i-voting technology may suffer from inflated estimations regarding the impact of trust in government on i-voting.

Although our study focuses on a country with an established i-voting system that is supported by major political parties across governments (Ehin et al. 2022), these findings highlight the importance of trust in government and technology for i-voting adoption elsewhere. The results suggest the relevance of generating confidence in the technology itself, how it is regulated and implemented, to ensure its success. In future work, we would encourage scholars to examine the role of citizens’ levels of trust in the governments and i-voting technology in other contexts. This is important as the way they are associated with turnout may differ in countries where internet voting has not been consistently implemented in the past years. Even though this study offers a cleaner perspective on the effect of trust on i-voting without interference of the novelty of the voting modality, the initial phase of introduction is also a crucial step in its implementation. Second, while the use of hypothetical scenarios has the clear advantage to allow random manipulation of trust, it is yet to be tested whether people who are less prone to support and use i-voting technologies could revisit their positions if this was promoted or implemented by their preferred, or an opposing, party. Our treatments simultaneously manipulated multiple aspects of trust in government or trust in technology. In line with this, the effects of these treatments in a real-world context, or of treatments that manipulate only one or a few of these aspects, have yet to be tested. Finally, while our measures of trust in i-voting technology focused on fraud susceptibility, as it is a crucial driving factor, other aspects of trust in i-voting technology might play a role too and should be further investigated.

Nonetheless, this paper shows crucial initial evidence that trust in government and trust in i-voting technology are separate and essential aspects in the study of i-voting participation. Their combined examination offers empirical, theoretical, and practical advantages to our understanding of democratic digitalization. As such, this may be an important step in the advancement of electoral participation, one of the most pressing issues in electoral democracies today.

## Supplemental Material

Supplemental Material - Trust in Government or in Technology? What Really Drives Internet Voting Supplemental Material for Trust in Government or in Technology? What Really Drives Internet Voting by M. Belén Abdala, Carolina Plescia, Ming M. Boyer, and Anna Lia Brunetti in Political Research Quarterly.

## Notes

1. Appendix A provides details about preregistration. The preregistration can be accessed here: https://osf.io/4avtz?view_only=713c4fcbc7474c77a6fc45a85dc94c28. The studies conducted in this paper received ethics approval by the University of Vienna.

2. This comparison is based on the most recent data on trust in government worldwide available for OECD as collected in 2019 (https://data-explorer.oecd.org/?lc=en&tm=DF_HSL_FWB&pg=0&snb=1). Specifically, trust in government is measured using the survey question: “In this country, do you have confidence in each of the following, or not? … How about national government?” The data shown reflect the percentage of respondents answering “yes” (rather than “no” or “don’t know”), which is 36.28 percent for the U.S. and 47.61 percent for the European countries included in the survey. These countries are: Austria, Belgium, Denmark, Estonia, Finland, France, Germany, Greece, Hungary, Iceland, Ireland, Italy, Latvia, Lithuania, Luxembourg, Netherlands, Norway, Poland, Portugal, Slovak Republic, Slovenia, Spain, Sweden, Switzerland, and United Kingdom. Information is sourced via the annual Gallup World Poll and the sample is ex ante designed to be nationally representative of the population aged 15 and over.

3. This comparison is based on Eurobarometer data for 2022. Trust in technology is measured using the survey question “How much trust do you have in certain media? For each of the following media, do you tend to trust it or tend not to trust it? The internet,” measured on a 1 (tend to trust)-2 (tend not to trust) scale. The level of trust in Estonia is 1.49 compared to 1.41 in EU-27 countries. While this difference is statistically significant, it is indeed quite modest (*p* = 0.001; diff = 0.08).

4. The proportion of respondents in our survey who have answered that they i-voted in the 2019 national election is approximately 15% points higher than the actual internet voting turnout. This proportion is in line with the estimates of most political surveys, in which the share of respondents who indicate they will vote is usually larger than that of the people who have voted (Selb and Munzert 2013). However, importantly, it is unlikely to affect inferences regarding the correlates of turnout, as shown by the work of (Achen and Blais 2015).

5. In each model, participants are excluded if they had incomplete answers, “don’t know,” “refuse to answer,” and “other” responses, as well as missing values in any variable in that specific model.

6. We repeated the analysis using the originally measured categorical variable in a multinominal model, excluding the postal option due to low selection numbers (*n* = 4). All analyses were replicated using this categorical operationalization (excluding the postal option), using multinomial models. Results are reported in the Appendix C (Table C4) and are consistent with those of the main analysis.

7. Although we offered an “other” option, the responses were selected by a very small number of participants. Consequently, we have excluded these respondents from this analysis.

8. All models were also run excluding two control variables that could be related to our dependent variables—trust in political parties and time spent online. The results, shown in Table C1, Appendix C are consistent with our main findings. Additionally, we conducted all models keeping the number of observations constant (*n* = 1263) which does not influence the results (Table C3, Appendix C).

9. Again, incomplete answers, “don’t know,” “refuse to answer,” and “other” responses in the outcome variables of the experimental module have been excluded from the analysis. Despite having to exclude a substantial set of participants, their number is very similar across the experimental conditions, as shown in Appendix B. While we conducted a power analysis during preregistration for *n* = 1,500, we had to test whether the current sample size provides the amount of power necessary to determine effects in the experiment. To do this, we performed a post-hoc sensitivity analysis (α = 0.05, η^2^ = 0.02, and *n* = 1,300), which indicates that we can reach a very high power of β = 0.97 and can reliably detect small effect sizes.

10. To carry out this analysis, the sample size had to be reduced, as 152 respondents answered don’t know or refused on the first indicator of acceptance and 232 on the second one. The sample size for the efficiency model is *n* = 1,148 and for the accountability model *n* = 1,068. More information available in Appendix G.
