# Supplementary material for: Trust in Government or in Technology? What Really Drives Internet Voting
Source: Polit Res Q. 2025 Feb 18;78(2):783–96. doi: 10.1177/10659129251321424 (PMC12037032; doi:10.1177/10659129251321424)
Supplement: Supplemental Material - Trust in Government or in Technology? What Really Drives Internet Voting [file sj-pdf-1-prq-10.1177_10659129251321424.pdf]

Online Appendix for "Trust in Government or in Technology? What Really Drives Internet Voting" by M. Belén Abdala, Carolina Plescia, Ming M. Boyer, and Anna Lia Brunetti in *Political Research Quarterly*.

Appendix A: Ethics and Pre-registration

Ethics approval

All the studies presented in this paper have received ethics approval from the University of Vienna's ethics board. Data collection was carried out by the agency Norstat and Norstat took care of any form of compensation for interviewees.

Consent to participate

Informed consent was obtained from all individual participants in the studies, who were also thoroughly debriefed after the experiment.

Pre-registration

Study 1 and Study 2 were conducted at the same time. The experiment carried out for this paper (Study 2) was preregistered in OSF prior to data collection (https://osf.io/4avtz?view_only=713c4fcbc7474c77a6fc45a85dc94c28). All the hypotheses and research questions that were preregistered in the framework of Study 2 were also used consistently for Study 1. It is important to mention, however, two small deviations from the pre-registration plan which established the expectation that "an increase in trust leads to an increase in online participation". The first deviation is meant to bring clarity in reflecting our pre-registered research question: we decided to divide the expectation in two hypotheses, each focusing respectively on higher levels of trust in government and higher levels of trust in the i-voting technology. The second deviation is meant to bring precision regarding the concepts

used: since our interest and focus is on predicting i-voting, we decided to specify i-voting as the outcome variable in the hypothesis instead of online participation. See Appendix D for a description of the experimental design.

Appendix B: General data quality

This section shows the data quality for both Study 1 and 2, as referred to in the main text. First, we describe how well the sample represents the national population of Estonia. Second, we focus on Study 2 and look into the sample distribution and drop-out rates at different stages of the experiment. Next, we examine the balanced distribution of respondents in each experimental condition (the trust/untrustworthy conditions for each Internet voting and Government dimensions of trust + a control group). Finally, we describe the distribution of age across groups, the only variable which show small imbalances.

Table B1. Data quality for the survey used in both Study 1 and 2

	Group	Quota (%)	Sample (%)
Gender	Men	46.6	46.13
	Women	53.4	53.4
Age Groups	18-24	8.4	8.13
	25-34	17.87	16.87
	35-44	17	15.47
	45-54	16.13	16
	55-64	16.33	14.33
	65+	24.27	20.6
Region	Põhja-Eesti	45.4	45.4
	Kesk-Eesti	9.2	9.2
	Kirde-Eesti	10.33	10.33
	Lõuna-Eesti	23.73	23.73
	Lääne-Eesti	11.33	11.33
Education	Low	17	13
	Mid	47	39.8
	High	36	47.2

Table B2. Sample distribution at different stages of the experiment (Study 2)

	Sample distribution						
**Experimental group**	**Treatment assignment**	**Pre-treatment intended voting modality**	**Manipulation check: trust in government**	**Manipulation check: trust in internet services**	**Post-treatment likelihood of voting in person (DV1)**	**Post-treatment likelihood of voting online (DV2)**	**Excluding all missing from both DVs**
Control	300	297	197	206	252	250	249
Trust i-voting	288	287	234	232	258	259	256
Distrust i-voting	306	305	264	263	275	275	271
Trust Government	316	309	258	261	278	280	276
Distrust Government	282	278	237	237	248	252	248
Total sample size	1492	1476	1190	1199	1311	1316	1300

Table B3. Descriptive statistics for each experimental group (Study 2)

			**Control**	**Internet Voting Technology**		**Government**		**Total**
				**Trust**	**Distrust**	**Trust**	**Distrust**	
Respondents								
Gender	Men	total	120.0	126.0	124.0	121.0	131.0	622.0
	Women	total	129.0	129.0	146.0	155.0	114.0	673.0
Age	Years	mean	45.75 (17.03)	46.32 (16.64)	48.79 (16.93)	50.07 (17.64)	47.16 (17.40)	47.68 (17.19)
Time spent online	Hours spent online	mean	5.11 (3.00)	5.28 (3.26)	5.18 (3.03)	5.13 (3.33)	5.33 (3.86)	5.20 (3.30)
Interest in politics	1= "very interest" to 4= "not at all interested"	mean	2.37 (0.86)	2.80 (0.87)	2.58 (0.81)	2.50 (0.89)	2.44 (0.89)	2.48 (0.87)
General propensity to trust	0= "you can't be too careful" to 10 ="most people can't be trusted"	mean	5.82 (2.41)	5.54 (2.70)	5.88 (2.40)	5.76 (2.43)	5.60 (2.48)	5.72 (2.49)
Education	Level of achieved education (from low to high)	mean	4.34 (1.92)	4.38 (1.97)	4.60 (1.89)	4.60 (1.87)	4.27 (1.85)	4.45 (1.90)
Subjective income	1="low" (finding it very difficult to manage on current income) to 4="high" (living comfortably on current income).	mean	2.73 (0.81)	2.71 (0.84)	2.73 (0.79)	2.66 (0.79)	2.65 (0.76)	2.70 (0.80)

Note: standard deviation in parenthesis. Differences in total values correspond to missing datapoints in each variable.

Table B4. Analysis of between group variance between experimental conditions (Study 2)

	Analysis of between group variance						
	**SS**	**df**	**MS**	**F**	**Prob > F**	**Bartlett's equal-variances test: chi2(4)**	**Prob>chi2**
Gender	1.38	4.00	0.34	1.38	0.24	0.02	1.00
Age	3382.88	4.00	845.72	2.88	0.02	1.12	0.89
Time spent online	9.33	4.00	2.33	0.21	0.93	21.19	0.01
Interest in politics	5.97	4.00	1.49	1.99	0.09	3.15	0.53
General propensity to trust	20.97	4.00	5.24	0.85	0.50	4.70	0.32
Subjective income	1.48	4.00	0.37	0.58	0.67	3.04	0.55

Table B5. Pairwise comparisons for age (Study 2)

	**Contrast**	**Std. Error**	**t**	**P>|t|**	**Tukey [95% conf. interval]**	
I-voting trust vs Control	0.57	1.53	0.37	1.00	-3.60	4.74
I-voting distrust vs Control	3.04	1.50	2.02	0.26	-1.07	7.15
Government trust vs Control	4.32	1.50	2.89	0.03	0.23	8.41
Government distrust vs Control	1.41	1.54	0.92	0.89	-2.79	5.61
I-voting distrust vs I-voting trust	2.47	1.49	1.66	0.46	-1.61	6.55
Government trust vs I-voting trust	3.75	1.49	2.52	0.09	-0.31	7.81
Government distrust vs I-voting trust	0.84	1.53	0.55	0.98	-3.33	5.01
Government trust vs I-voting distrust	1.28	1.47	0.87	0.91	-2.72	5.28
Government distrust vs I-voting distrust	-1.63	1.51	-1.08	0.82	-5.75	2.48
Government distrust vs Government trust	-2.91	1.50	-1.94	0.30	-7.01	1.18

Note. Calculated as Pairwise comparisons of the age distribution in each group, with Tukey effects.

Appendix C: Robustness checks of the cross-sectional results (Study 1)

This section presents four robustness checks of the analysis implemented for Study 1. First, we run all analyses excluding trust in political parties and time spent online as control variables, since these two variables could be related to our dependent variables. Results, presented in Table C1, are in line with the findings in the paper. Second, we run all analysis again including subjective income as a control variable. Results, presented in Table C2, are in line with the findings in the paper. Third, we run the same analysis as in the cross sectional, including only participants who provided valid responses for both key independent variables - trust in government and trust in i-voting (n=1263). Results are in line with the main analysis and are presented in Table C3. Fourth, we use the categorical operationalization of the dependent variable. Results are presented in Table C4. Probability to i-vote takes value 1 for people who prefer to vote in person on election day, 2 for those that prefer to vote in person before election day, and 3 for internet voting. Because of the categorical operationalization, we use multinomial models to estimate the effects of each type of trust over: the likelihood to vote in person before election day, and the likelihood to i-vote, separately, using the likelihood to vote in person on Election Day as the baseline, reference point.

Table C1. Predicting i-voting with trust in government and trust in i-voting, excluding trust in political parties and time spent online as controls.

	**Model 1**	**Model 2**	**Model 3**
Trust government	0.26***		0.04
	(0.02)		(0.03)
Trust i-voting		0.36***	0.35***
		(0.02)	(0.03)
Female	-0.18	-0.22	-0.22
	(0.13)	(0.15)	(0.15)
Age	-0.03	-0.02***	-0.02***
	(0.00)	(0.00)	(0.00)
Education	0.20	0.19***	0.19***
	(0.04)	(0.04)	(0.04)
Political interest	0.41	-0.39***	-0.41***
	(0.08)	(0.09)	(0.09)
Trust in political parties			
General propensity to trust	-0.05	-0.05	-0.06
	(0.03)	(0.03)	(0.03)
Time spent online (daily)			
Constant	1.13	0.13	0.08
	(0.29)	(0.34)	(0.34)
Observations	1396	1283	1272
LR chi2 (9)	268.12	478.76	476.68
Pseudo R2	0.1491	0.2904	0.2919

Notes: Standard errors in parentheses: * p<.05; ** p<.01; *** p<.001.

Table C2. Predicting i-voting with trust in government and trust in i-voting, using subjective income as a control

	Model 1	Model 2	Model 3	Model 4
Trust government	0.30***		0.04	0.06
	(0.03)		(0.03)	(0.04)
Trust i-voting		0.37***	0.35***	0.35***
		(0.02)	(0.03)	(0.03)
Female	-0.18	-0.23	-0.24	-0.25
	(0.13)	(0.15)	(0.15)	(0.16)
Age	-0.02***	-0.02***	-0.02***	-0.01***
	(0.00)	(0.00)	(0.00)	(0.00)
Education	0.17***	0.19***	0.21***	0.19***
	(0.04)	(0.04)	(0.04)	(0.04)
Political interest	-0.37***	-0.41***	-0.42***	-0.41***
	(0.08)	(0.10)	(0.09)	(0.10)
Trust in political parties	-0.11***	-0.02		-0.05
	(0.04)	(0.04)		(0.04)
General propensity to trust	-0.05*	-0.03	-0.05	-0.04
	(0.03)	(0.03)	(0.03)	(0.03)
Time spent online	0.07***	0.06**		0.06**
	(0.02)	(0.02)		(0.02)
Subjective Income	0.09	-0.07	-0.08	-0.09
	(0.09)	(0.10)	(0.10)	(0.10)
Constant	0.59	-0.15	0.21	-0.14
	(0.37)	(0.43)	(0.40)	(0.43)
Observations	1,358	1,247	1,251	1,242
LR chi2 (9)	269.83	465.15	465.77	467.46
Prob > chi2	0.0000	0.0000	0.0000	0.0000
Pseudo R2	0.1540	0.2907	0.2905	0.2932

Notes: Standard errors in parentheses: * p<.05; ** p<.01; *** p<.001.

Table C3. Predicting i-voting with trust in government and trust in i-voting

	Model 1	Model 2	Model 3	Model 4
Trust government	0.314***		0.04	0.0634
	(0.0304)		(0.03)	(0.0371)
Trust i-voting		0.365***	0.35***	0.343***
		(0.0227)	(0.03)	(0.0257)
Female	-0.223	-0.220	-0.22	-0.235
	(0.137)	(0.153)	(0.15)	(0.153)
Age	-0.0196***	-0.0146**	-0.02***	-0.0142**
	(0.00446)	(0.00485)	(0.00)	(0.00487)
Education	0.190***	0.183***	0.19***	0.174***
	(0.0387)	(0.0424)	(0.04)	(0.0428)
Political interest	-0.351***	-0.403***	-0.41***	-0.392***
	(0.0852)	(0.0951)	(0.09)	(0.0954)
Trust in political parties	-0.0934*	-0.00952		-0.0486
	(0.0388)	(0.0353)		(0.0423)
General propensity to trust	-0.0528	-0.0453	-0.06*	-0.0533
	(0.0294)	(0.0323)	(0.03)	(0.0327)
Time spent online	0.0661**	0.0612*		0.0604*
	(0.0226)	(0.0246)		(0.0247)
Constant	0.568	-0.312	0.08	-0.298
	(0.342)	(0.377)	(0.34)	(0.378)
Observations	1263	1263	1,272	1263
LR chi2 (9)	265.57	475.22	476.68	478.14
Log likelihood	-679.193	-574.37	-578.05879	-572.908
Pseudo R2	0.1635	0.2926	0.2919	0.2944

Notes: Standard errors in parentheses: * p<.05; ** p<.01; *** p<.001.

Table C4. Results from the multinomial logistic regression models.

**Pen and paper voting at a polling station before Election Day**	**Model 1**	**Model 2**	**Model 3**	**Model 4**
Trust government	0.0940*		0.07	0.102
	(0.0448)		(0.05)	(0.0533)
Trust i-voting		0.0130	-0.02	-0.0260
		(0.0351)	(0.04)	(0.0410)
Female	0.159	0.276	0.25	0.248
	(0.228)	(0.241)	(0.24)	(0.242)
Age	-0.00105	-0.00801	-0.01	-0.00751
	(0.00743)	(0.00782)	(0.01)	(0.00784)
Political interest	0.433**	0.382*	0.37**	0.405**
	(0.143)	(0.150)	(0.15)	(0.151)
Trust in political parties	-0.0303	-0.0135		-0.0671
	(0.0592)	(0.0530)		(0.0611)
General propensity to trust	-0.0184	0.0631	0.04	0.0527
	(0.0478)	(0.0505)	(0.05)	(0.0509)
Time spent online	-0.00677	-0.00184		0.000767
	(0.0396)	(0.0407)		(0.0408)
Education	0.0576	0.0930	0.09	0.0768
	(0.0633)	(0.0671)	(0.07)	(0.0679)
Constant	-2.818***	-2.767***	-2.83***	-2.789***
	(0.612)	(0.633)	(0.59)	(0.634)
**Online via Internet voting**	**Model 1**	**Model 2**	**Model 3**	**Model 4**
Trust government	0.332***		0.06*	0.0873*
	(0.0314)		(0.03)	(0.0392)
Trust i-voting		0.368***	0.34***	0.337***
		(0.0241)	(0.03)	(0.0274)
Female	-0.149	-0.157	-0.16	-0.175
	(0.141)	(0.162)	(0.16)	(0.163)
Age	-0.0217***	-0.0171***	-0.02***	-0.0160**
	(0.00460)	(0.00518)	(0.01)	(0.00523)
Political interest	-0.272**	-0.310**	-0.33***	-0.298**
	(0.0871)	(0.101)	(0.10)	(0.101)
Trust in political parties	-0.114**	-0.0175		-0.0654
	(0.0397)	(0.0371)		(0.0446)
General propensity to trust	-0.0530	-0.0313	-0.05	-0.0417
	(0.0299)	(0.0339)	(0.03)	(0.0345)
Time spent online	0.0673**	0.0610*		0.0611*
	(0.0233)	(0.0262)		(0.0264)
Education	0.190***	0.201***	0.21***	0.191***
	(0.0395)	(0.0450)	(0.05)	(0.0456)
Constant	0.685	-0.337	0.00	-0.373
	(0.352)	(0.398)	(0.36)	(0.401)
Observations	1385	1268	1,272	1263
LR chi2	300.28	489.56	493.23	495.96
Log Likelihood	-999.85162	-800.53357	-800.37079	-794.33465
Pseudo R2	0.1306	0.2342	0.2355	0.2379

Note. Standard errors in parentheses, * p<.05; ** p<.01; *** p<.001.

Appendix D: Study 2 - Full measurement and experimental design (Study 2)

This section presents the main information regarding the experimental design, preregistration of the experiment, debriefing materials and variables used for Study 2 (both manipulated and measured).

1. Information on the experiment design

Following the preregistration plan, participants were randomly allocated to one of five groups. Each group has exposed to only one of five descriptions of fictional country Mancosia, which are described below and followed the preregistration plan – accessed in OSF. Once the experimental module concluded, participants read a thorough debriefing, as shown in Text box D1.

The data were collected by panel agency Norstat between 3 and 17 January 2023 in Estonia, with the aim of reaching n=1,500. The experiment in the end had a sample size of n= 1,492 respondents. Each participant received a unique link to access the questionnaire. The survey company made all the decisions about compensation. The sample size was designed as part of a larger study and with the aim of having meaningful representation of the Estonian population (following a national representative sample on age, gender, region and education), and to detect small effects.

Text box D1. Debriefing materials

Important information

- Mancosia is a fictitious country.
- The statement that you saw does not reflect in any way the conditions of the e-government in Estonia or elsewhere.
- We use the information gathered from this exercise to investigate how trust impacts views of e-voting.

2. **Manipulated variables: experimental conditions regarding trust**

In the experiment, we expose participants to one of 5 descriptions of fictional country Mancosia. They are told to read the description to answer questions about the country afterward.

- Stimulus: control group

The next questions are about a country called Mancosia. Please read the following description of that country:

Mancosia is located in Europe with a territory of 83,871 km^2. In the last census of population in August 2007 Mancosia had 16,336,000 inhabitants. In the last years, the government has taken steps to implement e-government procedures, including internet voting and tax payments online.

- Stimulus: online voting system trustworthy group (I-voting Trust)

The next questions are about a country called Mancosia. Please read the following description of that country:

Mancosia is located in Europe with a territory of 83,871 km^2. In the last census of population in August 2007 Mancosia had 16,336,000 inhabitants. In the last years, the government has taken steps to implement e-government procedures, including internet voting and tax payments online.

Mancosia's legislation is **quite** transparent and its public authorities are **very** service-oriented and interested in supporting Mancosia's citizens. In particular, the government assigns a **large** budget to information technology. Such a **large** budget for information technology has **positive** repercussions on how much the citizens of Mancosia perceive e-government systems to be transparent, usable, secure and verifiable. In fact, in Mancosia online authentication for individuals to use e-government services **works** very well, and most people **trust** using an individual's unique personal identification number (PIN) for e-government services including internet voting and tax payment online. In Mancosia there is **a lot of trust** in a digital public administration and government.

- Stimulus: online voting system untrustworthy group (I-voting Distrust)

The next questions are about a country called Mancosia. Please read the following description of that country:

Mancosia is located in Europe with a territory of 83,871 km^2. In the last census of population in August 2007 Mancosia had 16,336,000 inhabitants. In the last years, the government has taken steps to implement e-government procedures, including internet voting and tax payments online.

Mancosia's legislation is **not** transparent and its public authorities are **not** service-oriented and **not** interested in supporting Mancosia's citizens. In particular, the government assigns a **small** budget to information technology. Such a **small** budget for information technology has **negative** repercussions on how much the citizens of Mancosia perceive e-government systems

to be transparent, usable, secure and verifiable. In fact, in Mancosia online authentication for individuals to use e-government services **does not work** very well, and most people **do not** trust using an individual's unique personal identification number (PIN) for e-government services including internet voting and tax payment online. In Mancosia there is **very little trust** in a digital public administration and government.

- Stimulus: government trustworthy group (Government Trust)

The next questions are about a country called Mancosia. Please read the following description of that country:

Mancosia is located in Europe with a territory of 83,871 km^2. In the last census of population in August 2007 Mancosia had 16,336,000 inhabitants. In the last years, the government has taken steps to implement e-government procedures, including internet voting and tax payments online.

Since Mancosia's autonomy in 1949, it has been marked with a **high** political stability and **a democratic** government. Referenda are **regularly** held, in which the citizens of Mancosia can co-decide on the legislation. The government enjoys a **good** reputation with the population. It can be concluded from opinion polls that 70% of the citizens are satisfied with the current government and more than 80% trust the institutions of the country. Moreover, according to an international corruption index (CPI), Mancosia is one of the European countries with the **lowest** levels of corruption. All these factors cause the citizens of Mancosia to **trust** their country's government a lot.

- Stimulus: government untrustworthy group (Government Distrust)

The next questions are about a country called Mancosia. Please read the following description of that country:

Mancosia is located in Europe with a territory of 83,871 km^2. In the last census of population in August 2007 Mancosia had 16,336,000 inhabitants. In the last years, the government has taken steps to implement e-government procedures, including internet voting and tax payments online.

Since Mancosia's autonomy in 1949, it has been marked with a **low** political stability and **an oligarchic (authority of few)** government. Referenda are **seldom** held, in which the citizens of Mancosia can co-decide on the legislation. The government enjoys a **bad** reputation with the population. It can be concluded from opinion polls that 70% of the citizens are **dis**satisfied with the current government and less than 30% trust the institutions of the country. Moreover, according to an international corruption index (CPI), Mancosia is one of the European countries with the **highest** levels of corruption. All these factors cause the citizens of Mancosia to **distrust** their country's government a lot.

3. **Measured variables**

- **Manipulation check (trust in government and trust in online services)**

Imagine please that you are a citizen of Mancosia and answer the following questions: [RANDOMIZED ITEMS]

How much trust would you have in the government of Mancosia?

How much trust would you have in the online services of the Mancosian government?

Scale from 0 (no trust at all) through 10 (complete trust)

- **Dependent variable 1: Likelihood to vote online or in person**

Imagine again that you are a citizen of Mancosia and that the country is holding elections in the upcoming weeks. Besides voting in person at a polling station, Mancosia allows online voting via Internet. [RANDOMIZED ITEMS]

How likely would you be to vote online via Internet in the election of Mancosia?

How likely would you be to vote in person at a polling station in the election of Mancosia?

Scale from 0 (very unlikely) through 10 (very likely)

- **Dependent variable 2: Online voting agreement**

On a 0-10 scale where 0 means strongly disagree and 10 means strongly agree, please state your level of agreement with the following statements: [RANDOMIZE ITEMS]

Online voting makes the administration of elections more efficient in Mancosia

The government of Mancosia can be held accountable for its actions through online voting

Scale from 0 (strongly disagree) through 10 (strongly agree)

- **Interest in politics (measured pre treatment exposure)**

Generally speaking, how interested are you in politics?

Very interested	1
fairly interested	2
a little interested	3
not at all interested	4

- **Time spent online (measured pre treatment exposure)**

On a typical day, about how much time do you spend using the internet on a computer, tablet, smartphone or other device, whether for work or personal use? Please give your answer in hours

- **General propensity to trust (measured pre treatment exposure)**

Generally speaking, would you say that most people can be trusted, or that you can't be too careful in dealing with people? Please answer on a 0 to 10 scale, where 0 means you can't be too careful and 10 means that most people can be trusted.

You can't be too careful	0
1	1
2	2
3	3
4	4
5	5
6	6
7	7
8	8
9	9
Most people can be trusted	10

Appendix E: Results for the manipulation check

This section presents the descriptives and main results regarding the manipulation check: measured as trust in government and trust in internet services.

Table E1. Summary of the manipulation checks

	Trust in government			Trust in online services		
**Experimental conditions**	**Mean**	**Std. dev.**	**Freq.**	**Mean**	**Std. dev.**	**Freq.**
Control	5.31	2.73	194	5.46	2.81	204
I-voting trust	6.02	2.82	226	6.29	2.90	225
I-voting distrust	1.74	2.25	253	1.81	2.23	252
Government trust	6.91	2.54	248	6.94	2.58	250
Government distrust	1.56	2.04	226	1.77	2.08	227
**Total**	4.27	3.36	1,147	4.38	3.39	1,158

Table E2. Effects of conditions over trust in government

					Tukey	
**Experimental conditions**	**Contrast**	**Std. Error**	**t**	**P>|t|**	**[95% conf. interval]**	
I-voting trust vs Control	0.71	0.24	2.91	0.03	0.04	1.37
I-voting distrust vs Control	-3.57	0.24	-15.07	0.00	-4.22	-2.92
Government trust vs Control	1.60	0.24	6.73	0.00	0.95	2.25
Government distrust vs Control	-3.75	0.24	-15.44	0.00	-4.42	-3.09

Note. Dependent variable is trust in government, measured on a scale from 0 (no trust at all) through 10 (complete trust).

Table E3. Effects of conditions over trust in online services

			Tukey			
**Experimental Conditions**	**Contrast**	**Std. Error**	**t**	**P>|t|**	**[95% conf. interval]**	
I-voting trust vs Control	0.83	0.24	3.41	0.01	0.16	1.50
I-voting distrust vs Control	-3.65	0.24	-15.32	0.00	-4.30	-2.99
Government trust vs Control	1.48	0.24	6.22	0.00	0.83	-0.11
Government distrust vs Control	-3.69	0.24	-15.13	0.00	-4.36	-3.02

Note. Dependent variable is trust in online services, measured on a scale from 0 (no trust at all) through 10 (complete trust).

Appendix F: Experimental results

This section presents the results of the experiment carried out using one-way ANOVAs and estimating the effects of each experimental condition as contrasts over: 1. the likelihood to i-vote, and 2. the likelihood to vote in person. Additionally, we present the results of the interaction effect of trust in the i-voting technology and trust in government on the inclination to i-vote or vote in person. The interaction effects are calculated as ANOVAS between the level of trust (low vs high) and the type of trust (i-voting technology vs. government) on each dependent variable. The control condition is excluded from this analysis.

Table F1. Summary of the effects of each condition of the voting experiment over the likelihood to i-vote, as a one-way ANOVA.

	**Mean**	**Std. dev.**	**Freq.**
Control	5.26	3.86	249
I-voting trust	6.05	3.83	256
I-voting distrust	1.92	2.72	271
Government trust	6.45	3.61	276
Government distrust	2.88	3.49	248
Total	4.52	3.95	1,300

Note: Likelihood to vote online measured on a 0-10 scale as dependent variable

Analysis of variance

**Source**	**SS**	**df**	**MS**	**F**	**Prob>F**
Between groups	4260.59	4	1065.1476	86.12	0.000
Within groups	16016.003	1295	12.36757		
Total	20276.593	1299	15.609387		

Bartlett's equal-variances test: chi2(4) = 38.97

Prob>chi2=0.000

Table F2. Estimated effects of each experimental condition over the likelihood to i-vote

			Unadjusted		Tukey			
DV: likelihood to vote online	**Contrast**	**Std. err.**	**t**	**P>t**	**t**	**P>t**	**[95% conf. Interval]**	
I-voting trust vs Control	0.79	0.31	2.54	0.01	2.54	0.08	-0.06	1.65
I-voting distrust vs Control	-3.34	0.31	-10.81	0.00	-10.81	0.00	-4.18	-2.49
I-voting distrust vs I-voting trust	-4.13	0.31	-13.48	0.00	-13.48	0.00	-4.97	-3.29
Government trust vs Control	1.19	0.31	3.88	0.00	3.88	0.00	0.35	2.03
Government distrust vs Control	-2.37	0.32	-7.52	0.00	-7.52	0.00	-3.24	-1.51
Government distrust vs Government trust	-3.57	0.31	-11.59	0.00	-11.59	0.00	-4.41	-2.73
Government trust vs I-voting trust	0.40	0.31	1.31	0.19	1.31	0.69	-0.44	1.23
Government distrust vs I-voting trust	-3.17	0.31	-10.11	0.00	-10.11	0.00	-4.02	-2.31
Government trust vs I-voting distrust	4.53	0.30	15.06	0.00	15.06	0.00	3.71	5.35
Government distrust vs I-voting distrust	0.96	0.31	3.12	0.02	3.12	0.02	0.12	1.81

Note: Effects calculated as pairwise comparisons of means unadjusted and with equal variances, using Tukey's adjustment. The dependent variable is the likelihood to i-vote.

Table F3. Summary of the effects of the voting experiment over the likelihood to vote in person, as a one-way ANOVA.

	**Mean**	**Std. dev.**	**Freq.**
Control	5.73	3.65	249.00
I-voting trust	4.98	3.85	256.00
I-voting distrust	6.72	3.66	271.00
Government trust	5.54	3.69	276.00
Government distrust	5.42	3.97	248.00
Total	5.69	3.80	1300.00

Note: Likelihood to vote in person measured on a 0-10 scale as dependent variable.

Analysis of variance

**Source**	**SS**	**df**	**MS**	**F**	**Prob>F**
Between groups	441.892292	4	110.473073	7.80	0.00
Within groups	18346.1777	1295	14.1669326		
Total	18788.07	1299	14.4634873		

Bartlett's equal-variances test: chi2(4) = 2.8002

Prob>chi2= 0.592

Table F4. Estimated effects of each experimental condition over the likelihood to vote in person.

			Unadjusted		Tukey			
	Contrast	Std. err.	t	P>t	t	P>t	[95% conf.	Interval]
I-voting trust vs Control	-0.75	0.34	-2.23	0.03	-2.23	0.17	-1.66	0.17
I-voting distrust vs Control	0.99	0.33	3.00	0.00	3.00	0.02	0.09	1.89
I-voting distrust vs I-voting trust	1.74	0.33	5.30	0.00	5.30	0.00	0.84	2.63
Government trust vs Control	-0.19	0.33	-0.59	0.55	0.55	0.98	-1.09	0.70
Government distrust vs Control	-0.31	0.34	-0.92	0.36	-0.92	0.89	-1.23	0.61
Government distrust vs I-voting trust	0.43	0.34	1.30	0.20	1.30	0.69	-0.48	1.35
Government trust vs I-voting trust	0.55	0.33	1.69	0.09	1.69	0.44	-0.34	1.44
Government distrust vs I-voting distrust	-1.30	0.33	-3.94	0.00	-3.94	0.00	-2.21	-0.40
Government trust vs I-voting distrust	-1.19	0.32	-3.69	0.00	-3.69	0.00	-2.07	-0.31
Government distrust vs Government trust	-0.12	0.33	-0.35	0.72	-0.35	0.997	-1.02	0.78

Note: Effects calculated as pairwise comparisons of means unadjusted and with equal variances, using Tukey's adjustment. The dependent variable is the likelihood to vote in person.

Table F5. Two-way ANOVA with the interaction effect between the level and type of trust on the likelihood to vote online.

Source	Partial SS	df	MS	F	Prob>F
Model	4092.24	3.00	1364.08	115.86	0.00
Level of trust	3885.58	1.00	3885.58	330.04	0.00
Type of trust	121.76	1.00	121.76	10.34	0.00
Level*Type	20.99	1.00	20.99	1.78	0.18
Residual	12326.45	1047.00	11.77		
Total	16418.69	1050.00	15.64		
			Observations		1,051
			Root MSE		3.4312
			R-squared		0.2492
			Adjusted R-squared		0.2471

Table F6. Two-way ANOVA with the interaction effect between the level and type of trust on the likelihood to vote in person.

Source	Partial SS	df	MS	F	Prob>F
Model	441.38	3.00	147.13	10.24	0.00
Level of trust	172.50	1.00	172.50	12.00	0.00
Type of trust	37.08	1.00	37.08	2.58	0.11
Level*Type	225.80	1.00	225.80	15.71	0.00
Residual	15045.21	1047.00	14.37		
Total	15486.58	1050.00	14.75		
			Observations		1051.00
			Root MSE		3.79
			R-squared		0.03
			Adjusted R-squared		0.03

Appendix G

This section presents the results of an additional exploration. We replicated the experimental analysis using an additional outcome variable that captures the level of acceptance of i-voting. The variable, scaled on a 0-10 scale, measures the level of agreement with the extent to which i-voting allows for efficiency and accountability. Higher values represent higher levels of agreement with the following statements: "Online voting makes the administration of elections more efficient in Mancosia" and "The government of Mancosia can be held accountable for its actions through online voting". The order of the items was randomized. The analysis was conducted using one-way ANOVAS and contrasts comparing each treatment condition to the control group.

Table G1. Effects of the treatment conditions on the level of acceptance of i-voting, measured as the extent to which i-voting is efficient (on a 0 "strongly disagree" to 10 "strongly agree" scale).

Experimental conditions	Mean	Std. Dev.	Freq.
Control	6.60	3.41	226.00
I-vote trust	7.09	3.39	234.00
I-vote distrust	3.76	3.37	233.00
Government trust	7.14	3.01	249.00
Government distrust	4.07	3.38	206.00
Total	5.79	3.62	1148.00

Analysis of variance

	SS	df	MS	F	Prob>F
Between groups	2566.70	4.00	641.68	58.66	0.00
Within groups	12503.44	1143.00	10.94		
Total	15070.14	1147.00	13.14		

Bartlett's equal-variances test: chi2(4)= 5.31

Prob>chi2= 0.257

Estimated effects of each condition over perceptions of efficiency.

	Contrast	Std. err.	t	Tukey P>t	Tukey [95% conf. Interval]	
I-voting trust vs Control	0.50	0.31	1.61	0.49	-0.35	1.34
I-voting distrust vs Control	-2.83	0.31	-9.18	0.00	-3.68	-1.99
Government trust vs Control	0.54	0.30	1.79	0.38	-0.29	1.37
Government distrust vs Control	-2.53	0.32	-7.94	0.00	-3.40	-1.66
I-voting distrust vs I-voting trust	-3.33	0.31	-10.88	0.00	-4.17	-2.49
Government trust vs I-voting trust	0.05	0.30	0.15	1.00	-0.78	0.87
Government distrust vs I-voting trust	-3.03	0.32	-9.58	0.00	-3.89	-2.16
Government trust vs I-voting distrust	3.38	0.30	11.20	0.00	2.55	4.20
Government distrust vs I-voting distrust	0.30	0.32	0.96	0.87	-0.56	1.17
Government distrust vs Government trust	-3.07	0.31	-9.86	0.00	-3.92	-2.22

Note: Effects calculated as pairwise comparisons of means with equal variances, using Tukey's adjustment. The dependent variable is the efficiency, measured on a 0-10 scale to the following statement: "Online voting makes the administration of elections more efficient in Mancosia".

Table G2. Effects of the treatment conditions on the level of acceptance of i-voting, measured as the extent to which i-voting allows for accountability (on a 0 "strongly disagree" to 10 "strongly agree" scale).

Experimental conditions	Mean	Std. Dev.	Freq.
Control	4.96	3.40	204.00
I-vote trust	5.11	3.28	207.00
I-vote distrust	2.83	2.91	233.00
Government trust	5.47	3.24	217.00
Government distrust	3.11	3.09	207.00
Total	4.27	3.36	1068.00

Analysis of variance

	SS	df	MS	F	Prob>F
Between groups	1310.24	4.00	327.56	32.35	0.00
Within groups	10763.17	1063.00	10.13		
Total	12073.41	1067.00	11.32		

Bartlett's equal-variances test: chi2(4)= 6.1187

Prob>chi2= 0.190

Estimated effects of each condition over perceptions of accountability

	Contrast	Std. err.	Tukey t	P>t	Tukey [95% conf. Interval]	
I-voting trust vs Control	0.15	0.31	0.48	0.99	-0.71	1.01
I-voting distrust vs Control	-2.12	0.31	-6.96	0.00	-2.96	-1.29
Government trust vs Control	0.51	0.31	1.64	0.47	-0.34	1.36
Government distrust vs Control	-1.84	0.31	-5.88	0.00	-2.70	-0.99
I-voting distrust vs I-voting trust	-2.27	0.30	-7.48	0.00	-3.10	-1.44
Government trust vs I-voting trust	0.36	0.31	1.16	0.77	-0.49	1.20
Government distrust vs I-voting trust	0.36	0.31	1.16	0.77	-0.49	1.20
Government trust vs I-voting distrust	2.63	0.30	8.77	0.00	1.81	3.45
Government distrust vs I-voting distrust	2.63	0.30	8.77	0.00	1.81	3.45
Government distrust vs Government trust	-2.35	0.31	-7.62	0.00	-3.20	-1.51

Note: Effects calculated as pairwise comparisons of means with equal variances, using Tukey's adjustment. The dependent variable is the efficiency, measured on a 0-10 scale to the following statement: "The government of Mancosia can be held accountable for its actions through online voting".

Figure G1. Effects of being exposed to each "trust" condition over the level of acceptance of *i*-voting.

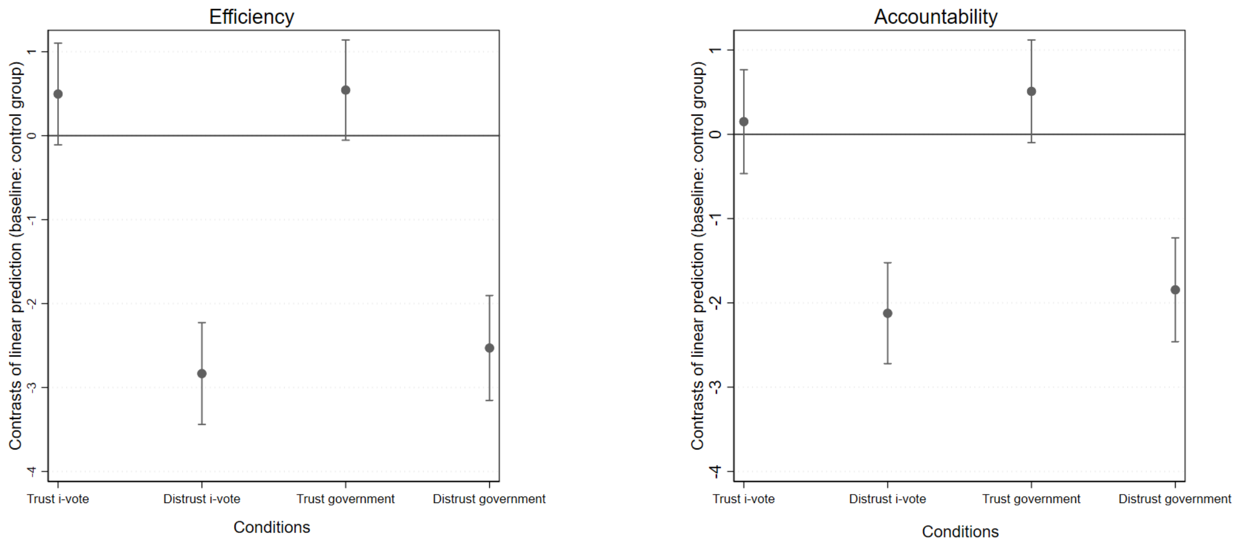

Note: The outcome variables are the level of agreement with the efficiency and accountability provided through i-voting, measured on a 0 "very strongly disagree" to 10 "strongly agree" scale. The effects are calculated as contrasts. The control group is used as baseline. The lines indicate 95% confidence intervals. Estimates are obtained from the ANOVA models presented in Appendix G.
